# A renewable approach to electric vehicle charging through solar energy storage

**DOI:** 10.1371/journal.pone.0297376

**Published:** 2024-02-29

**Authors:** Muhammad Umair, Nabil M. Hidayat, Ahmad Sukri Ahmad, Nik Hakimi Nik Ali, M. I. Mohd Mawardi, Ezmin Abdullah

**Affiliations:** 1 School of Electrical Engineering, College of Engineering, Universiti Teknologi MARA, Shah Alam, Selangor, Malaysia; 2 Battery Energy Storage Technology Laboratory (BEST), College of Engineering, Universiti Teknologi MARA, Shah Alam, Selangor, Malaysia; 3 Petronas Research Sdn. Bhd., Bangi Government and Private Training Centre Area, Bandar Baru Bangi, Selangor, Malaysia; Vardhaman College of Engineering, INDIA

## Abstract

Developing novel EV chargers is crucial for accelerating Electric Vehicle (EV) adoption, mitigating range anxiety, and fostering technological advancements that enhance charging efficiency and grid integration. These advancements address current challenges and contribute to a more sustainable and convenient future of electric mobility. This paper explores the performance dynamics of a solar-integrated charging system. It outlines a simulation study on harnessing solar energy as the primary Direct Current (DC) EV charging source. The approach incorporates an Energy Storage System (ESS) to address solar intermittencies and mitigate photovoltaic (PV) mismatch losses. Executed through MATLAB, the system integrates key components, including solar PV panels, the ESS, a DC charger, and an EV battery. The study finds that a change in solar irradiance from 400 W/m^2^ to 1000 W/m^2^ resulted in a substantial 47% increase in the output power of the solar PV system. Simultaneously, the ESS shows a 38% boost in output power under similar conditions, with the assessments conducted at a room temperature of 25°C. The results emphasize that optimal solar panel placement with higher irradiance levels is essential to leverage integrated solar energy EV chargers. The research also illuminates the positive correlation between elevated irradiance levels and the EV battery’s State of Charge (SOC). This correlation underscores the efficiency gains achievable through enhanced solar power absorption, facilitating more effective and expedited EV charging.

## Introduction

The rapid transition towards sustainable energy is only possible with a large-scale proliferation of Electric Vehicles (EVs) [1, 2]. EVs offer a low cost over the lifecycle and an environmentally friendly alternative to traditional fossil fuel vehicles. EVs have much lower carbon footprints than fuel cells or conventional transport options. However, the widespread adoption of EVs requires developing a simple and robust charging infrastructure, specifically Direct Current (DC) chargers, to ensure efficiency, reliability, and accessibility [3]. Unlike Alternating Current (AC) chargers, DC chargers provide low-loss, efficient, and fast charging to modern electric transportation. However, installing many chargers on the already saturated power grid is not feasible. Therefore, DC chargers with renewable energy as the prime input source have emerged as a sustainable alternative. Renewable energy sources, predominantly solar energy, are an innovative approach to EV charging [4, 5]. Solar energy, harnessed from the sun, offers an abundant and clean power source, presenting an optimal solution for sustainable EV charging [6]. However, solar intermittencies and photovoltaic (PV) losses are a significant challenge in embracing this technology for DC chargers.

On the other hand, the Energy Storage System (ESS) has also emerged as a charging option. When ESS is paired with solar energy, it guarantees clean, reliable, and efficient charging for EVs [7, 8]. This combination liberates EV owners from relying solely on traditional grid power while contributing to the environment and developing self-sufficiency in energy consumption. Integrating solar energy, ESS, and DC charging involves notable challenges in research and development, particularly concerning compatibility and the management of energy flows [9]. The proposed system promotes sustainability and encourages decentralized energy generation, enabling consumers to control their energy needs.

Recent literature lays the groundwork for the current research; many articles address solar energy, EVs, and charging infrastructure [10]. However, most of these works focus on individual components, overlooking the potential of an integrated system [11]. Research on DC fast chargers often discusses the technical requirements for efficient EV charging. Still, very few studies have coupled renewable energy sources to it. Similarly, studies on ESS generally concentrate on-grid applications, overlooking their pivotal role in a decentralized, renewable energy setup for EVs [7]. A few studies have examined integrating solar power and ESS in EV charging systems. Still, these often lack a comprehensive approach that includes DC chargers, PV-induced losses, energy management, and automation, thus leaving a gap in the literature [12, 13].

In a PV system, a mismatch loss occurs when the PV modules in the system are not identical. Larger PV arrays are prone to more mismatch losses. The mismatch losses are accessed as a difference between the theoretical power of perfectly matched PV modules and the measured PV power output [14]. The differences can be due to manufacturing differences, shading, soiling, and temperature. Mismatch losses in PV panels can occur for various reasons, such as electrical connections, misalignment of PV cells, top surface polishing/dirt, orientation differences, or different temperature coefficients [15]. The PV cell manufacturing process is complex and involves several steps. Ensuring identical doping and other manufacturing-related issues is challenging, which can lead to cell-level mismatches [16]. These mismatches are further enhanced when PV panels are connected in a PV array. A percentage of a PV array output power represents mismatch losses. For example, if an array has a mismatch loss of 0.1% with the PV array output of 1000 W, then the mismatch loss would be 0.001 kW (0.1% x 1000 W). Quantifying the mismatch loss is important because it significantly contributes to system losses.

This research aims to bridge this gap by integrating these elements and optimizing the resulting system for maximum efficiency and sustainability. This research significantly contributes to the growing renewable energy and electric mobility field. Through design and integration, the study establishes a robust and efficient system without needing the power grid, combining solar energy, ESS, and efficient charging solutions tailored for EVs. It provides insights into a self-sustaining energy system. It lays the foundation for future innovations in sustainable transportation and energy management. This work extends beyond academic research by offering a practical and environmentally responsible solution to EV charging, signifying a new era in energy utilization.

## Methods

### The overall framework

**Fig 1:**
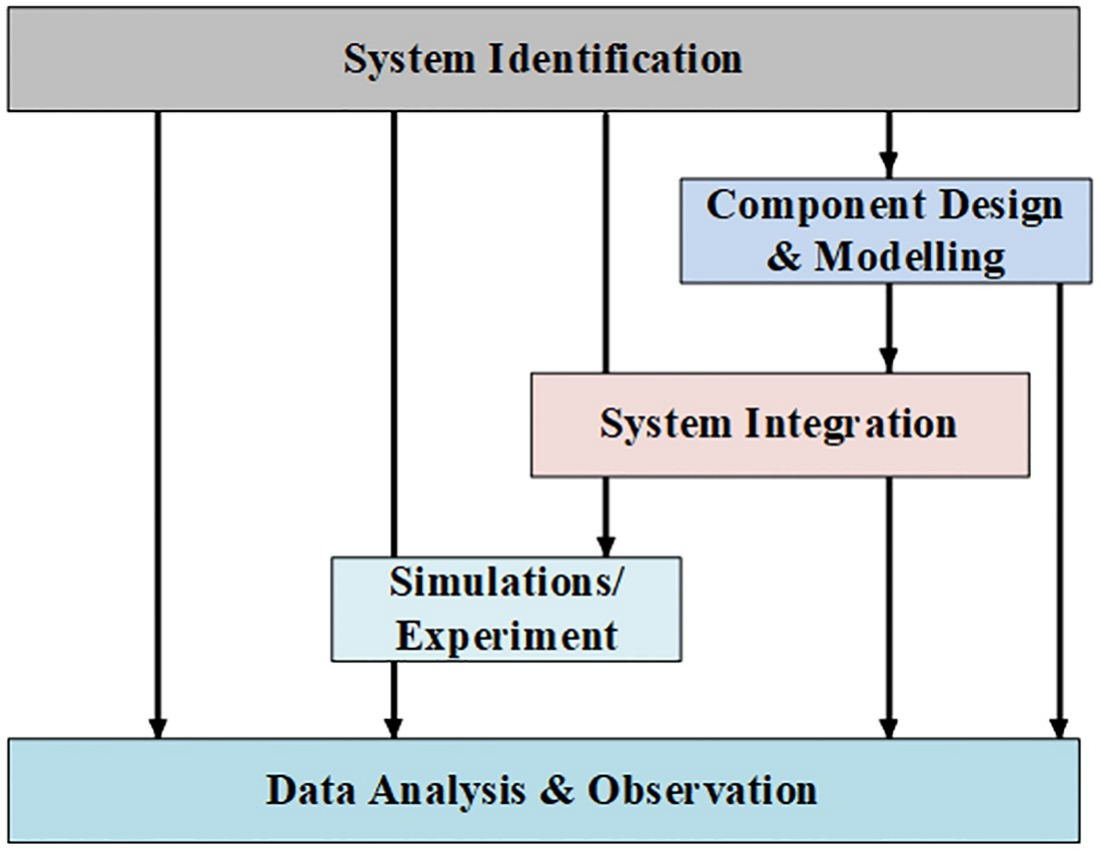
The overall framework of system.

Fig 1 presents the overall framework of this study. The framework started with identifying the components of the novel PV-ESS integrated system. Component identification was followed by individual component design and modeling. Next, the designed subsystems were coupled together for an overall system. Next, simulations were conducted on the integrated system, and finally, the results were analyzed. The subsequent step involved comprehensive data analysis upon successfully integrating all the individual elements. The study provided observed insights, which have significantly contributed to the system’s overall capabilities and potential applications.

### System elements and integration

**Fig 2:**
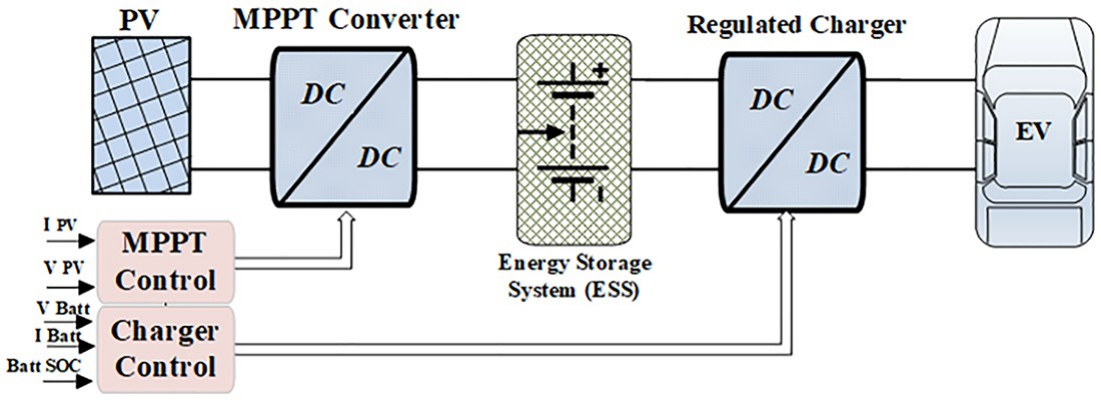
System elements and integration.

Fig 2 shows the proposed system projecting a solar energy harvesting and storage architecture for EVs. The primary components of this system include a PV array, a Maximum Power Point Tracking (MPPT) front-end converter, an energy storage battery, and the charging DC-DC converter. The system manages intermittent factors such as partial shading and PV mismatch losses, ensuring optimal energy harnessing into the ESS battery by dynamically adjusting the operational point of the PV system to maximize power transfer [17]. Furthermore, the batteries of the EVs are combined to simulate a practical application of charging EVs.

Lithium-ion batteries are widely used in EVs and ESSs due to their high energy density, prolonged lifespan, and cost-effectiveness. However, an intelligent Battery Management System (BMS) is often required to measure and control various battery attributes during these system’s charge/discharge cycles. Measuring the lithium-ion State of Charge (SOC) is challenging because of its unexpectedly highly time-varying behavior, electrochemical properties, and complexity. SOC represents the extent to which the battery has been charged or discharged, which assesses the operational status and remaining life.

MATLAB provides detailed battery models that encompass the dynamics of lithium-ion batteries. These models integrate various parameters, including voltage, current, temperature, and capacity, enabling a comprehensive representation of the battery’s behavior. By considering the non-linear and time-variant nature of the battery, the model can provide accurate estimations of SOC under diverse operating conditions. This level of accuracy is crucial for optimizing charging and discharging strategies, enhancing the overall efficiency of EVs and ESSs, and ensuring the longevity of the lithium-ion battery.

The two main stages methodically carried out the design process of the simulation model. First, the process modeled each element individually, considering its electrical characteristics and performance parameters. This meticulous modeling captured the unique attributes of each component accurately. Second, the process unified the entire system into a comprehensive circuit. This integration carefully considered the interactions and connections between the components, ensuring seamless and efficient overall system functionality.

### Simulation characteristics

The impact of elevated radiation and temperature variation on the output of PV panels and their subsequent effect on the SOC of EV batteries was simulated using MATLAB. A series of interconnected blocks resulted in a dynamic model of a proposed EV charger with solar energy as a primary source. The "Irradiance Source Block" in MATLAB facilitated inputting the time-varying irradiance profile. In the simulation, a general PV profile that depicts a typical solar day was utilized. The duration of daylight hours in a typical solar day varies depending on geographical location, time of year, and local weather conditions. It is important to note that while the sun may be above the horizon for about 12 hours, the intensity of sunlight is not constant throughout the day. Peak sun hours represent the hours during which the sun’s intensity is sufficient for solar panels to operate at maximum efficiency. A reasonable assumption is considering 5 to 6 peak sun hours per day for preliminary simulation. Non-peak sun hours represent the lower solar intensity and reduced energy production.

The simulation also utilized a "Temperature Source Block" to include the effects of varying temperatures on PV modules. Temperature variations significantly affect PV efficiency. However, the energy buffering property of the ESS of the proposed system makes the EV charging unaffected due to these variations. "PV Array Block" offered comprehensive modeling features. This block models the array’s response under varying irradiance and temperature conditions, thus facilitating an understanding of the effect of high radiation on PV output and analysis mismatch losses.

Moreover, a separate "Mismatch Model Block" in MATLAB implemented the mathematical model of mismatch losses. The "MPPT Controller Block" implemented the simulations’ maximum power point tracking perturb & observe (P&O) algorithm. It adjusts the operational point of the PV system to optimize power output. The simulation analysis also used the "Data Logging Block," which records all the time-varying variables, e.g., PV voltage/current, charging voltage/ current, output power, solar irradiance, and EV battery’s SOC. These variables are also displayed through the "Scope Block".

### System parameters

The simulation model incorporated the JKM380M-72-V solar module by Jinko Solar Co., Ltd, chosen for its high-efficiency rate and compatibility with other system components. The solar irradiance was investigated within a range of 400 W/m^2^ to 1000 W/m^2^ with the incremental rate of 50 W/m^2^, maintaining a constant temperature setting of 25°C. Conversely, the effect of temperature on the ESS output power was studied at temperatures of 5°C, 15°C, 25°C, 35°C, and 45°C. The detailed parameters of this solar module are presented in Table 1.

**Table 1:** Parameters for solar panels.

Parameters	Values
Maximum Power (W)	380.295
Cells per module (Ncell)	72
Open circuit voltage Voc (V)	48.9
Short-circuit current Isc (A)	9.75
Voltage at maximum power point Vmp (v)	40.5
Current at maximum power point Imp (A)	9.39
Temperature coefficient of Voc (%/deg.C)	-0.326
Temperature coefficient of Isc (%/deg.C)	0.055005

**Fig 3:**
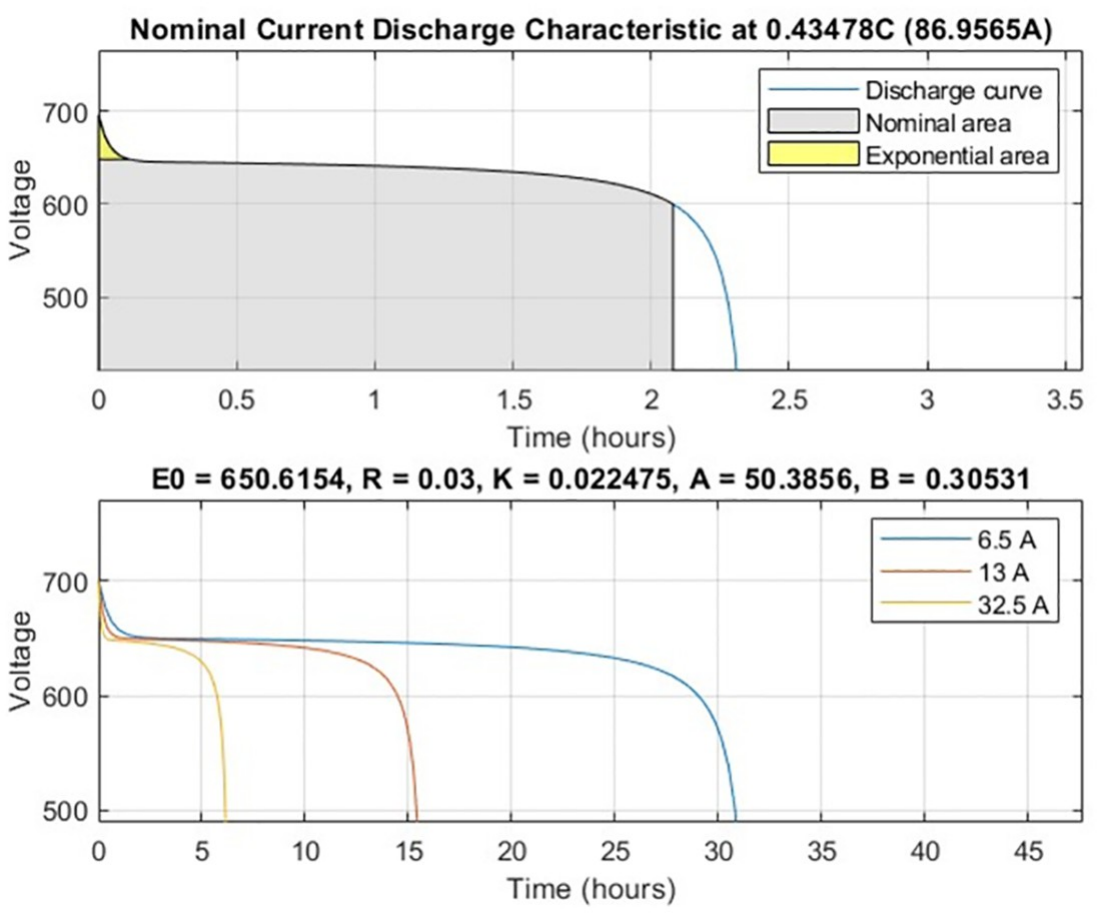
Discharge curve for ESS.

On the other hand, a lithium-ion battery was used for ESS, possessing a nominal voltage of 600 V and a rated capacity of 200 Ah. The battery’s initial SOC was set at 100%, and the response time was calibrated to 1 second. The discharge curve for the ESS battery is observed in Fig 3.

**Fig 4:**
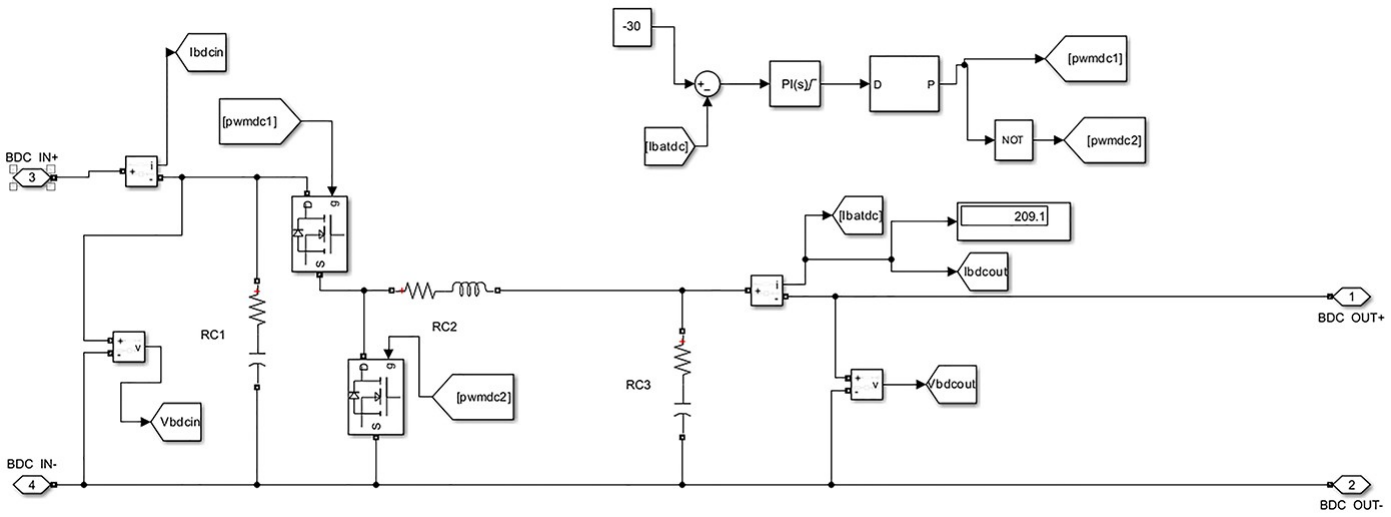
Buck-boost converter.

Moreover, the simulation assumed a 6 kW PV and a 30 kW DC charger with a DC output voltage of 200–700 V DC and an output current of 100 A. A high-capacity charger was utilized to mimic the fast charging behavior. It also considered parameters for the EV battery, including a battery capacity of 40 kWh, a voltage of 350 V, and a battery energy of 114 Ah. The buck-boost converter circuit, a critical part of the model, is shown in Fig 4.

These specifications contribute to the system’s accurate energy flow and conversion emulation, reflecting the real-world interactions between the components.

Table 2 summarizes the overall system parameters used in the simulations, calculations, and discussions.

**Table 2:** Integrated PV-ESS DC charger parameters.

Parameters	Values
ESS Battery Type	Lithium-ion
ESS Nominal Voltage	600 V
ESS Rated Capacity	200 Ah or 120kW
Initial SOC for ESS Battery	100%
ESS Response Time	1 second
PV Array Power	6 kW
DC Charger Power	30 kW
DC Charger Output Voltage Range	200–700 V DC
DC Charger Max Output Current	100 A
EV Battery Capacity	40 kWh
EV Battery Voltage	350 V
EV Battery Energy	114 Ah

### Mathematical calculation

To estimate the charging time of an ESS from a PV array, the battery capacity is calculated using the following equation.


$$ESS\;Battery\;Capacity=\frac{ESS\;voltage\;\left(V\right)\;\times \;Rated\;Capacity\;(Ah)\;}{1000}=\frac{600\;\times \;200}{1000}=120\;kWh$$ (1)

The charging time for ESS is:

$$Charging\;Time\;for\;ESS=\frac{ESS\;Battery\;Capacity}{PV\;Charging\;Power}=\frac{120\;kWh}{6\;kW}=20\;hrs$$ (2)

A fully discharged ESS battery would require approximately 20 hours to recharge using a 6 kW PV array, resulting in the ESS SOC reaching 100%. The charging time for an EV battery using a 600 V and 200 Ah ESS is determined by the power supplied by the DC charger and the power the EV battery can accept. The EV voltage and current constrain the power, hence the formula for charging power is:

ChargingPower=min.(DCChargerPower,EVBatteryVoltage×ChargerMaximumCurrent) (3)

$$Charging\;Power=min.\;\left(30\;kW,\;350\;V\;\times \;100\;A\right)=\;30\;kW$$ (4)

The EV charging time can now be calculated as follows:

$$Charging\;Time\;for\;EV=\frac{EV\;Battery\;Capacity}{Charging\;Power}=\frac{40\;kWh}{30\;kW}=1.33\;hrs$$ (5)

For the designed system using the 6 kW PV array, the estimated charging time for the ESS would be approximately 20 hours. In comparison, it would take about 1.33 hours to charge the EV battery with a battery capacity of 40 kWh using the 30 kW DC charger with a voltage range of 200–700 V and a maximum output current of 100 A.

## Result and discussion

### Impact of solar irradiance on the output power of solar panels

**Fig 5:**
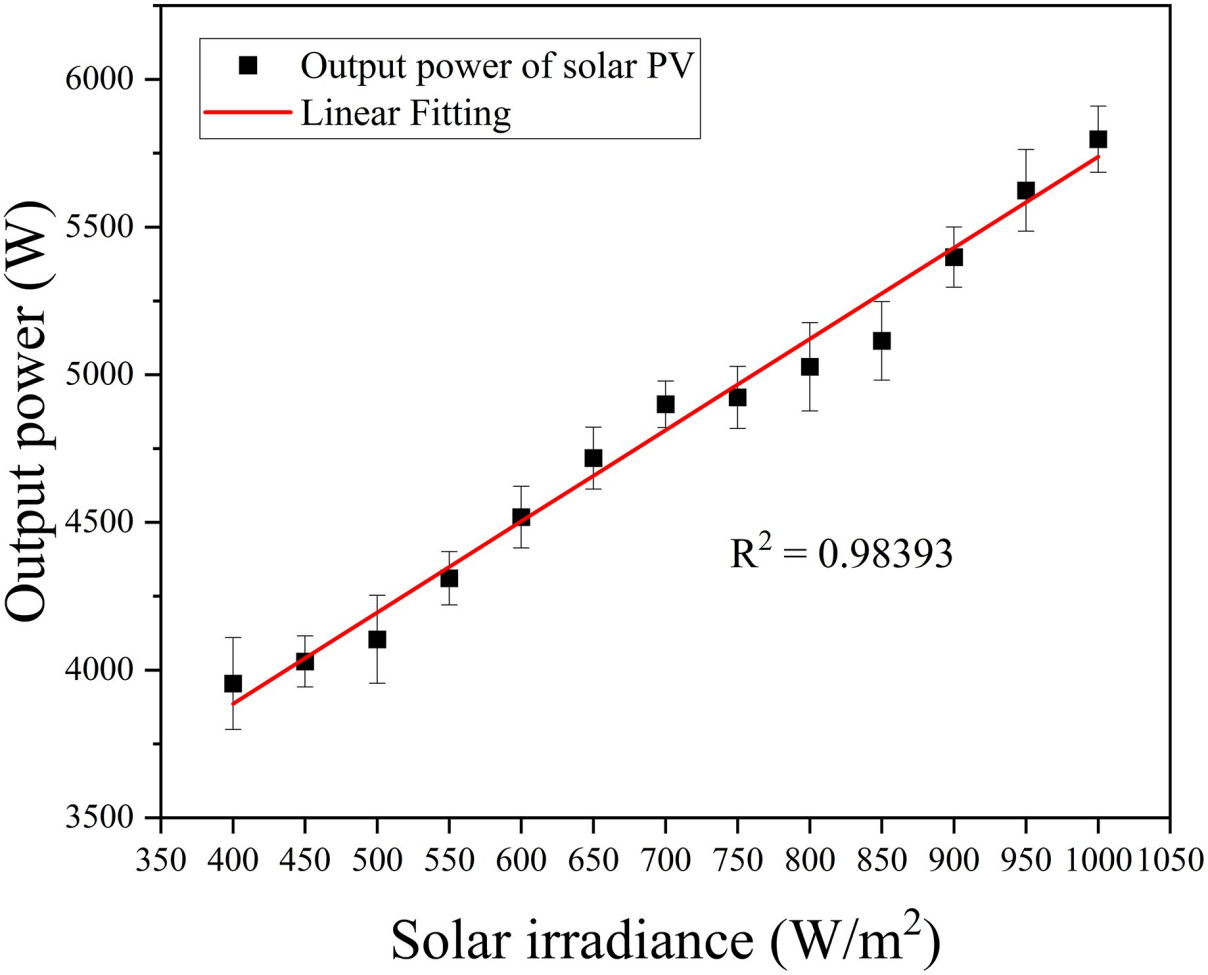
Output power of solar PV.

Solar irradiance is the amount of solar power received per unit area, which varies based on several influencing factors such as geographic location, time of day, seasonal changes, and atmospheric conditions [18, 19]. The findings reveal that the solar panel output power increases linearly by 47% when the solar irradiance increases from 400 W/m^2^ to 1000 W/m^2^. The relationship between solar irradiance and output power demonstrated a linear correlation, with an R^2^ value of 0.98393, as depicted in Fig 5. This result underscores the necessity of installing solar panels in locations exposed to the highest irradiance levels.

The direct correlation between the increase in output power and higher solar irradiance reflects the underlying principle that an abundance of sunlight enhances power output [18]. In contrast, a decrease in irradiance produces the opposite effect. Since a more significant number of photons striking the solar cells induces more electrons into motion, generating a higher current and increased power [20]. Furthermore, since solar cells are sensitive to specific wavelengths, alterations in the spectral composition of sunlight can influence operational efficiency [21].

Though the study consistently maintains the temperature at 25°C throughout, it’s essential to recognize that more sunlight often corresponds to increased temperatures. This can be significant as solar cells tend to become less efficient at higher temperatures, a condition that might offset the advantages gained from increased irradiance [22].

A noteworthy observation from the study is identifying the solar panel’s output power at an irradiance of 1000 W/m^2^ and a temperature of 25, whereby the results showed the maximum output power of 5799 W, with an output voltage of 311.2 V and an output current of 18.78 A. These specific values further elucidate the tangible relationship between solar irradiance and the performance characteristics of solar panels.

### Energy storage system for EV charging

#### SOC of energy storage system

**Fig 6:**
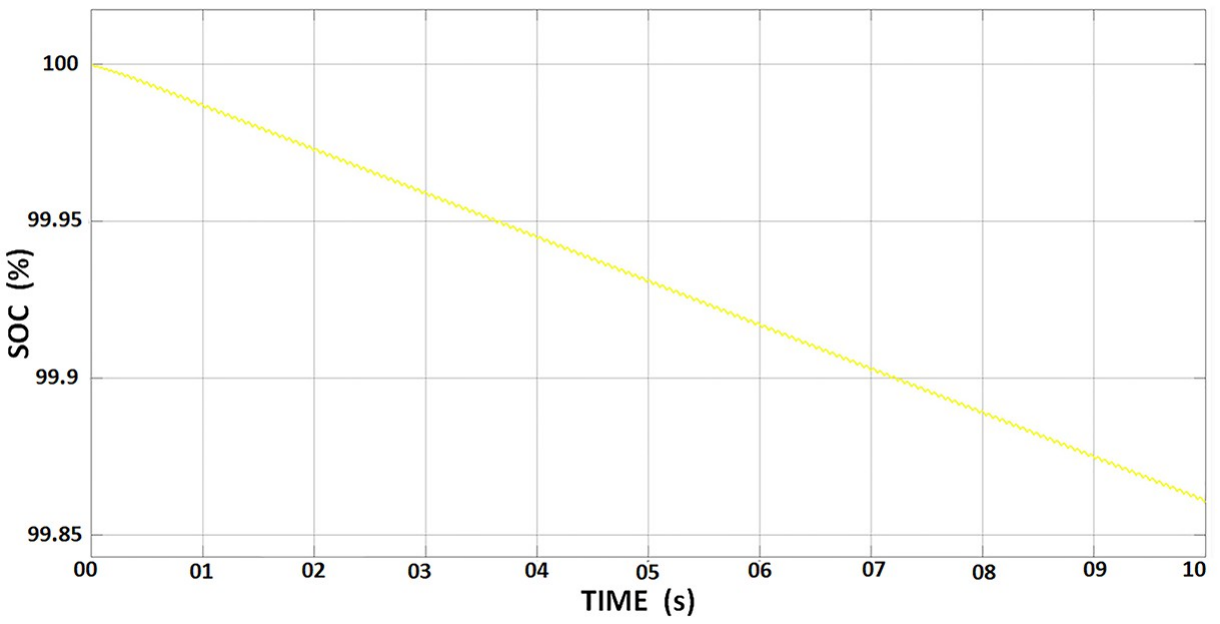
State of Charge (SOC) curve of ESS.

After capturing energy from the solar panel, the MPPT system channels the power before sending it to the ESS for storage. Since solar panel output fluctuates due to variations in sunlight intensity, temperature, and other environmental conditions, operating at peak efficiency is impossible. The MPPT rectifies this by continuously monitoring and adjusting the solar panels’ voltage and current to perform at the maximum power point [23]. After optimization by the MPPT, the output power increased to 6.45 kW, a growth of 11.2%, with a solar irradiance of 1000 W/m^2^ and solar temperature of 25°C. However, despite this optimization, the SOC of the ESS declined slightly from 100% to 99.86%, as shown in Fig 6.

This 0.14% drop in SOC of ESS could be attributed to minor losses during the conversion process with DC-DC converters used in MPPT to align the voltage levels of the solar panel and battery [24]. Even though these losses are marginal, they may account for a slight decrease in the ESS’s SOC. The solar panel, MPPT, and ESS establish a seamless system that captures and stores solar energy as efficiently as possible, ensuring its availability for future use. This integration is crucial to the overall effectiveness and efficiency of the solar energy system.

#### Impact of solar irradiance and temperature on ESS performance

**Fig 7:**
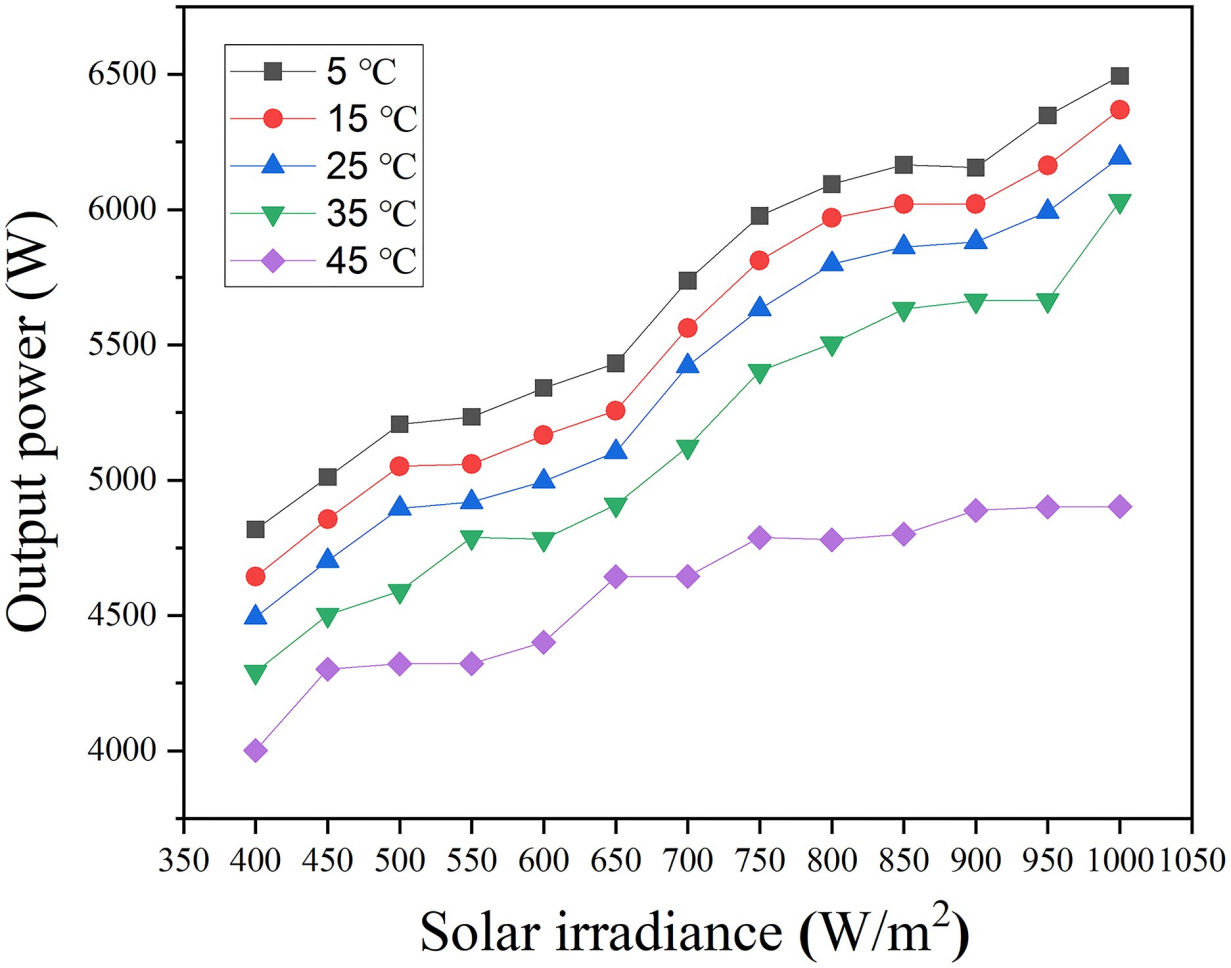
Output power of ESS with solar irradiance levels.

Fig 7 presents the relationship between solar irradiance and the average output power of the ESS at solar temperatures of 5°C, 15°C, 25°C, 35°C, and 45°C. The observation shows that as solar irradiance increases, the output power of ESS also increases for all solar temperatures. This correlation stems from the corresponding increase in the solar panel’s output power, as presented in Fig 5. Contrarily, the SOC of the ESS remains consistently at 99.85% across all tested solar irradiance values. This consistency indicates that the system operates at total capacity, with the ESS being 100% fully charged at the defined solar irradiance levels. Therefore, any increase in solar irradiance does not lead to a corresponding increase in the SOC of ESS, signifying that the system efficiently utilizes the available energy without exceeding its storage capacity.

Moreover, the observation shows that the increase in solar irradiance from 400 W/m^2^ to 1000 W/m^2^ leads to a 38% increase in the average output power of the ESS at room temperature of 25°C, which is 9% lower than the average output power increase in solar panels. This discrepancy might be due to the MPPT’s potentially reduced effectiveness at higher irradiance levels, causing losses during conversion. Additional contributing factors might include battery charging efficiency, voltage matching, conversion losses, and potential system limitations [22, 25]. Collectively, these elements can result in a situation where an increase in solar irradiance does not translate to a proportional increase in stored energy in the ESS.

Furthermore, data in S2 Table suggests that the average output power decreases across all irradiance levels as solar temperature rises. This reduction is linked to the diminished ability of the solar panel to convert sunlight into electrical energy at elevated temperatures [26]. Multiple factors contribute to this efficiency loss as the temperature rises. One key factor is the reduction in the open-circuit voltage of the solar cells within the panel, which directly affects output power [27]. Moreover, higher temperatures can trigger thermal annealing in the panels, leading to a drop in efficiency and increasing the risk of physical stress [28]. This stress can compromise the panel’s mechanical integrity and potentially cause micro-cracks, further reducing efficiency.

For the ESS, the average output power at 5°C shows a 24% increase when solar irradiance increases from 400 W/m^2^ to 1000 W/m^2^. Conversely, at 45°C, the average output power for the ESS also increases by 13%. However, the rate of increase in the average output power at 45°C is lower than at 5°C. This discrepancy is because higher temperatures result in slower charging speeds due to the solar panel generating less power [29]. This, in turn, leads to a slower charging rate for both the ESS and EVs.

Consequently, less electrical energy would be available for storage in the ESS, possibly necessitating supplementary power from other sources. Although increased irradiance can boost output power, this gain may be offset by losses incurred at higher temperatures. Specific solar panel characteristics, including its temperature coefficient, influence the net effect.

#### Impact of solar irradiance levels on electric vehicle charging

Table 3 presents the simulation results for the average output power, ranging from a solar irradiance of 400 W/m^2^ to 1000 W/m^2^, with an average error of 127.84 W, at the solar temperature of 25°C. An observation from the data reveals that the SOC of the EV increases by 11.05% when the solar irradiance reaches 1000 W/m^2^. This increase occurs because higher solar irradiance enables the solar system to absorb more sunlight, consequently boosting the electrical energy produced by the solar panels. With the increased sunlight, the MPPT identifies a new maximum power point, extracting more power from the solar panels. This augmented energy is then transferred to the ESS, serving as a reservoir for the energy. The EV is assumed to be connected within this system, permitting the DC charger to draw the accumulated energy from the ESS, efficiently transmitting it to the EV’s battery. More energy is generated and stored at higher solar irradiance levels, so more power is available for EV battery charging. As a result, the SOC of the EV battery rises in proportion to the energy conveyed to it. This enhanced SOC not only reflects the charging state of the EV but also illustrates the responsiveness of the system to variations in solar irradiance.

**Table 3:** Simulation results at room temperature of 25°C.

Solar irradiance (W/m^2^)	Average output power (kW)	Error (W)	SOC of EV (%)	EV Status
400	4.49	124.1813	79.6%	Charging
450	4.7	123.391	80.1%	Charging
500	4.89	135.9203	80.6%	Charging
550	4.91	119.6007	80.9%	Charging
600	4.99	124.976	82.2%	Charging
650	5.1	134.3962	82.8%	Charging
700	5.42	112.7697	82.8%	Charging
750	5.63	133.9739	84.4%	Charging
800	5.79	146.6026	85.2%	Charging
850	5.86	133.2529	86.0%	Charging
900	5.88	124.4843	86.0%	Charging
950	5.99	124.1813	87.6%	Charging
1000	6.19	124.1813	88.4%	Charging

## Limitations

While the study offers an in-depth, simulation-based analysis of an integrated solar system for EV charging, it is not without its limitations. The research predominantly employs MATLAB simulations to gauge the system’s performance. Although helpful in modeling ideal conditions, these simulations might not capture the variability encountered in real-world scenarios due to environmental, operational, and material factors not considered in the simulation.

Additionally, the study sets a fixed temperature of 25°C to assess the impact of solar irradiance on the EV battery. This overlooks the need for a more nuanced examination of system performance under weather conditions like rain, cloud cover, or differing humidity levels. These omissions could result in a mismatch between the simulated and actual efficiency of the solar panels and, by extension, the whole system. The study is also circumscribed by its focus on solar irradiance levels ranging between 400 W/m^2^ and 1000 W/m^2^ without exploring the effects of other solar panels or varying battery states for the ESS. Similarly, it does not examine the impact of deep discharges or cyclic loading on long-term battery health. Another limitation is the assumption of constant efficiency for electrical components, such as DC-DC converters and the MPPT system. These components’ efficiencies can fluctuate based on operational conditions, affecting the system’s overall performance.

Furthermore, the research assumes a consistent efficiency for the MPPT system without accounting for potential variances caused by changing solar irradiance or other operational circumstances. It restricts its scope to lithium-ion batteries for both the ESS and EV without exploring the possible advantages or drawbacks of using alternative battery types, which might be more cost-effective or environmentally sustainable. The study also sidesteps economic considerations in implementing and maintaining such an integrated system, including solar panels, ESS, converters, and other hardware costs.

## Conclusion

Novel EV chargers and comprehensive energy strategies emerge as a technological imperative and a pivotal step toward shaping a more sustainable and interconnected future. EV chargers need an omnipresent charging infrastructure and universal compatibility through faster, grid-independent charging solutions. Overcoming these problems is crucial for widespread EV adoption. This paper introduces an innovative PV-ESS integrated system to improve EV fast charging. The proposed system addresses solar intermittencies by redirecting excess solar energy to an ESS. This stored energy is efficiently utilized during EV charging, minimizing grid dependency and mitigating mismatch losses. MPPT is implemented in a front-end converter, optimizing the PV array utility. At the same time, a second-stage DC-DC converter regulates the EV charging process. A hypothetical charging scenario is also provided where a 6 kW solar panel charges a 200 Ah ESS. The same ESS can charge a 40 kW EV within 1.33 hours. The research findings highlight a direct correlation between increased solar irradiance and elevated output power from solar panels, signifying the solar panel placement for maximum utility. Furthermore, the study reveals an improvement in EV charging efficiency corresponding to increased solar irradiance. Specifically, a step change from 400 W/m^2^ to 1000 W/m^2^ results in a 47% increase in average solar panel output power; similarly, the ESS registers a corresponding 9% lower growth. The study finds a direct proportionality between solar irradiance and the EV battery’s SOC.

This approach exemplifies sustainable practices by utilizing renewable energy to diminish reliance on fossil fuels and curb greenhouse gas emissions. Nonetheless, transitioning from conventional grid-based charging to this solar-based model presents challenges to existing power grid infrastructures, requiring extensive modification and potentially leading to transient stability issues. The limitations of this study necessitate further research and development to transition from simulation to practical implementation. Future work may involve detailed testing under varied environmental conditions, integrating existing grid infrastructure, and exploring hybrid charging solutions combining solar and renewable sources. The potential benefits of this system in contributing to a more sustainable and resilient energy landscape make it a promising avenue for future exploration and refinement.

## Supporting information

S1 Table Output power of solar PV with irradiance levels from 400 to 1000 W/m^2^. (XLSX)

S2 Table Output power of ESS with solar irradiance levels from 400 to 1000 W/m^2^ under different solar temperature conditions. (XLSX)
